# Associations of calcium and magnesium intakes and their intake ratio with albuminuria in middle-aged and older adults

**DOI:** 10.1371/journal.pone.0335412

**Published:** 2025-11-26

**Authors:** Keiko Kabasawa, Michihiro Hosojima, Kazutoshi Nakamura, Ribeka Takachi, Norie Sawada, Shoichiro Tsugane, Junta Tanaka, Suguru Yamamoto, Yumi Ito

**Affiliations:** 1 Department of Health Promotion Medicine, Niigata University Graduate School of Medical and Dental Sciences, Asahimachi-dori, Chuo-ku, Niigata, Japan; 2 Division of Clinical Nephrology and Rheumatology, Kidney Research Center, Niigata University Graduate School of Medical and Dental Sciences, Asahimachi-dori, Chuo-ku, Niigata, Japan; 3 Department of Clinical Nutrition Science, Kidney Research Center, Niigata University Graduate School of Medical and Dental Sciences, Asahimachi-dori, Chuo-ku, Niigata, Japan; 4 Division of Preventive Medicine, Niigata University Graduate School of Medical and Dental Sciences, Asahimachi-dori, Chuo-ku, Niigata, Japan; 5 Department of Food Science and Nutrition, Nara Women’s University Graduate School of Humanities and Sciences, Kitauoya-Higashimachi, Nara, Japan; 6 Division of Cohort Research, National Cancer Center Institute for Cancer Control, Tsukiji, Chuo-ku, Tokyo, Japan; 7 International University of Health and Welfare Graduate School of Public Health, Akasaka, Minato City, Tokyo, Japan; International University of Health and Welfare, School of Medicine, JAPAN

## Abstract

Calcium and magnesium both work together and against each other by sharing homeostatic regulatory systems in the kidneys. Previous studies suggested that these minerals and their intake ratio were associated with chronic health conditions such as cardiovascular disease. Our objective was to investigate the association of calcium and magnesium intake and their intake ratio with albuminuria. This cross-sectional study analyzed data from a Japanese community-based cohort comprising 6,849 individuals aged ≥40 years (mean age 68.8 years, 51.3% women). Energy-adjusted dietary intake of calcium and magnesium and the calcium-to-magnesium intake ratio were derived from a validated food frequency questionnaire. The outcome was spot urinary albumin-to-creatinine ratio (ACR, mg/g). Linear and logistic regression analyses were performed with adjustments for potential confounders. The analytic population had 1.65 and 73.5 mL/min/1.73 m^2^ as median intake ratio and estimated glomerular filtration rate, respectively. In multivariable linear regression analysis, lower intakes of calcium and magnesium were associated with the log-transformed ACR but, after mutual adjustment, only calcium intake was independently associated with the ACR (regression coefficient −0.084; 95%CI, −0.149, −0.019). A lower intake ratio was associated with the log-transformed ACR (regression coefficient, −0.085; 95%CI, −0.150, −0.021). These associations were evident overall and in male participants. Although magnesium intake was associated with albuminuria only before accounting for calcium intake, calcium intake and calcium-to-magnesium intake ratio demonstrated robust associations. Our findings support that the importance of calcium intake and its balancing with magnesium might extend to kidney health, especially albuminuria.

## Introduction

Calcium and magnesium work together and against each other, with calcium intake affecting magnesium retention in humans and vice versa. Both minerals are essential for maintaining enzymatic systems and their function in neuromuscular contraction, bone formation, and blood coagulation. In the kidneys, a major part of calcium and magnesium regulation takes place in consecutive segments from the proximal tubule to the connecting duct where filtered calcium and magnesium are reabsorbed and excreted [1,2]. Albuminuria is not only a strong risk factor for cardiovascular disease [3], but also a multifactorial marker of kidney damage, including tubulointerstitial alteration [4]. Calcium and magnesium intakes have been linked to many aspects of cardiovascular health [5–9]. However, these observations are inconclusive, with suggestions that their effects on health are outcome-specific and modified by the intake ratio [10,11].

We hypothesized that the intakes of calcium and magnesium and the calcium-to-magnesium intake ratio are associated with albuminuria. Secondarily, we examined the interaction of intake ratio on the relationship of calcium and magnesium intakes with albuminuria.

## Materials and methods

### Study population

This cross-sectional study used baseline data on individuals with available urine samples in the Uonuma Chronic Kidney Disease (CKD) cohort study, a population-based prospective cohort study investigating lifestyle-related and clinical risk factors for CKD. The baseline study has been detailed elsewhere [12]. Written informed consent was obtained from all participants. At baseline (2012–2015), 43,211 residents were enrolled in the questionnaire survey (residents aged 40 years or older in the cities of Minamiuonuma and Uonuma and the town of Yuzawa in Niigata Prefecture, Japan). Of these, 6,942 residents consented to provide urine samples. For this specific study, we excluded those with missing information on estimated glomerular filtration rate (eGFR, n = 1), drinking habit (n = 1), and exercise habit (n = 1). Those who had an extremely high or low energy intake (> or < 3 standard deviations [SD] from the mean, n = 90) were excluded, leaving 6,849 residents as the final study sample. The study protocol was approved by the Ethics Committee of Niigata University (2017−0054).

### Dietary assessment

Nutrients and food groups were assessed by the self-administered food frequency questionnaire (FFQ) from the Japan Public Health Center-based Prospective Study for the Next Generation [13]. This questionnaire assesses the average consumption of 172 food and beverage items during the past year estimated by nine frequency categories and three amounts for each meal. Intakes of energy, calcium, magnesium, and the remaining nutrients and food groups were calculated using the Standard Tables of Food Composition in Japan 2010 [14]. A previous validation study revealed Spearman’s rank correlation coefficients for energy-adjusted calcium and magnesium intakes based on the FFQ and 12-day weighed food records as 0.58 and 0.39 in men and 0.42 and 0.51 in women, respectively [15]. Using these energy-unadjusted values, we calculated the calcium-to-magnesium intake ratio as calcium intake divided by magnesium intake. When we reported respective nutrients, minerals, and food groups, they were adjusted for energy intake using the residual method [16].

### Urine sample measurements

A spot urine sample was collected during a baseline health checkup. Urinary albumin and creatinine concentrations were measured using fresh urine by the latex agglutination and enzymatic methods, respectively. We assessed albuminuria as the urinary albumin-to-creatinine ratio (ACR) and defined microalbuminuria as an ACR ≥ 30 mg/g and high normoalbuminuria as an ACR ≥ 10 mg/g. We also explored the relationship between the spot urinary calcium-to-magnesium ratio and albuminuria because the intake of these minerals alters their renal excretion [1,2]. Of the analyzed population, urinary calcium and magnesium in the stored frozen urine sample could be measured in 6,282 participants using the methylxylenol blue and xylidyl blue methods, respectively.

### Covariates

We used the baseline health checkup data, which included blood tests and measurements of body weight, height, and systolic and diastolic blood pressures. Smoking status (current smoker or not), drinking habit (never or rarely drinking or not), exercise habit (regular exercise habit or not), and history of urinary tract stone were self-reported by each participant. Body mass index (BMI) was calculated as measured body weight (kg) divided by measured body height (m) squared. Serum creatinine was measured using the enzymatic method, and eGFR was calculated by an equation for Japanese adults: eGFR (mL/min/1.73 m^2^) = 194 × (serum creatinine [mg/dL])^(−1.094)^ × age (years)^(−0.287)^ (× 0.739 [for women]) [17]. Diabetes was defined as HbA1c ≥ 6.5%, fasting plasma glucose ≥126 mg/dL, casual plasma glucose ≥200 mg/dL, or use of antidiabetic medication. Hypertension was defined as systolic blood pressure ≥140 mmHg, diastolic blood pressure ≥90 mmHg, or use of antihypertensive medication.

### Statistical analysis

In the present study, we imputed a value of 0.5 mg/g (approximately half the limit) for ACR in 81 participants who had ACR below the limit of detection. Participant characteristics are summarized as mean ± SD, median (interquartile interval [IQI]), or number (percentage) according to the quartiles of calcium and magnesium intake and their intake ratio, respectively. We tested the differences in the prevalence of microalbuminuria according to quartiles of calcium and magnesium intakes and their intake ratio by using the chi-squared test. The correlation between calcium and magnesium intakes and the calcium-to-magnesium intake ratio was assessed by using Spearman’s correlation coefficients. Locally weighted scatter plot smoothing (LOWESS) curves were applied to visualize the relationship of calcium and magnesium intakes and the intake ratio with albuminuria.

Multivariable linear regression analysis was used to estimate the associations of calcium and magnesium intakes and their intake ratio (independent variables) with albuminuria (a dependent variable). In this analysis, we used the natural logarithm for these independent variables and the ACR due to their skewed distributions. Model 1 was adjusted for sex, age, survey area (three areas, a dummy variable), current smoker, never or rarely drinking, regular exercise habit, and energy intake (quartile by sex). Model 2 was further adjusted for BMI, diabetes, hypertension, history of urinary tract stone, and eGFR. Model 3 was performed to account for each mineral to assess whether their associations with albuminuria were mutually independent. We used similar multivariable logistic regression models with quartiles of calcium and magnesium intakes or the intake ratio as independent variables and microalbuminuria (ACR ≥ 30 mg/g) or high normoalbuminuria (ACR ≥ 10 mg/g) as a dependent variable. We set the highest quartile as a reference and tested *P* for trend values by treating the quartile of each mineral and intake ratio as ordinal variables.

We further evaluated the modifying effects of the intake ratio on the associations of calcium and magnesium intakes with albuminuria. We applied multivariable linear regression analysis between calcium or magnesium intake and albuminuria (Model 3) according to groups divided by the ratio levels at each median value and assessed an interaction cross-term between calcium or magnesium intake and the ratio group.

Next, we repeated the multivariable linear regression analysis (Model 3) in subgroups and evaluated an interaction cross-term between an exposure and subgroup by age (<vs. ≥ 70 years), diabetes status, hypertension status, and kidney function (eGFR < vs. ≥ 60 mL/min/1.73 m^2^). As a sensitivity analysis, we performed similar linear and logistic regression analyses, excluding participants who used calcium or magnesium supplements (Models 1, 2, and 3). Furthermore, we performed these analyses using the urinary calcium-to-magnesium ratio instead of the intake ratio (Models 1 and 2). Models were further adjusted for fasting or not.

Data were accessed for research purposes on April 30, 2024. Statistical analyses were performed using SAS version 9.4 (SAS Institute Inc., Cary, NC).

## Results

The study population comprised 51.3% women (n = 3,515) and had a mean (SD) age of 68.8 (9.9) years and eGFR of 74.3 (15.8) mL/min/1.73 m^2^. The median energy-adjusted intakes of calcium and magnesium and their intake ratio were 567.9 (IQI, 419.7–746.4) mg/day, 326.1 (IQI, 279.8–381.1) mg/day, and 1.65 (IQI, 1.30–2.06), respectively. The median ACR was 11.7 (IQI, 6.0–25.0) mg/g and microalbuminuria was observed in 21.4%. Compared with participants in the other three quartile groups, those in the lowest quartile of calcium intake were more likely to be younger, male, current smokers, and hypertensive and to have a history of urinary tract stone and higher eGFR and were less likely to never or rarely drink and to have an exercise habit (Table 1). Characteristics of participants in the lowest quartiles of magnesium intake (Table 1) and the calcium-to-magnesium intake ratio (S1 Table) were largely similar to those in the lowest quartile of calcium intake. The prevalence of microalbuminuria was likely to differ across quartiles of magnesium intake (*P*-value = 0.080) but not across those of calcium intake and the intake ratio (S2 Table).

**Table 1:** Descriptive characteristics according to quartiles of dietary calcium and magnesium intakes.

	Quartile of energy adjusted intake^a^			
**Dietary calcium intake, mg/day**	≥746.4	567.9–746.3	419.7–567.8	<419.7
N	1,712	1,712	1,713	1,712
Energy-unadjusted calcium, mg/day	761.4 [537.3, 1323.1]	579.9 [436.6, 775.5]	471.7 [339.3, 649]	292.4 [187.8, 441.7]
Energy-unadjusted magnesium, mg/day	341.3 [246.1, 456.7]	326.1 [251.3, 422.6]	314.8 [238.3, 409.6]	252.9 [178.3, 348.4]
Calcium-to-magnesium intake ratio	2.39 [1.94, 3.23]	1.81 [1.57, 2.04]	1.53 [1.32, 1.75]	1.17 [0.96, 1.4]
Urinary albumin-to-creatinine ratio, mg/g	12 [7, 25]	12 [7, 25]	11 [6, 26]	10.8 [6, 25]
Age, years	70.6 ± 9.3	69.6 ± 9.3	68.5 ± 9.8	66.6 ± 10.9
Male sex, n (%)	614 (35.9)	642 (37.5)	864 (50.4)	1,214 (70.9)
Body mass index, kg/m^2^	22.6 ± 3.0	22.6 ± 3.1	22.7 ± 3.1	22.8 ± 3.0
eGFR, mL/min/1.73 m^2^	73.3 ± 15.5	73.1 ± 15.0	74.4 ± 15.5	76.5 ± 16.7
Current smoker, n (%)	129 (7.5)	151 (8.8)	252 (14.7)	421 (24.6)
Never or rarely drinking, n (%)	1028 (60.1)	950 (55.5)	781 (45.6)	500 (29.2)
Regular exercise habit, n (%)	692 (40.4)	693 (40.5)	608 (35.5)	539 (31.5)
Diabetes, n (%)	195 (11.4)	151 (8.8)	155 (9.1)	175 (10.2)
Hypertension, n (%)	834 (48.7)	874 (51.1)	895 (52.3)	943 (55.1)
History of urinary tract stone, n (%)	68 (4.0)	75 (4.4)	84 (4.9)	92 (5.4)

**Dietary magnesium intake, mg/day**	≥381.1	326.1–381.0	279.8–326.0	<279.8
N	1,712	1,713	1,711	1,713
Energy-unadjusted calcium, mg/day	632.1 [444.2, 883.5]	576.6 [410.8, 800.5]	506.4 [357.1, 717.3]	336.5 [212.6, 517.4]
Energy-unadjusted magnesium, mg/day	397.1 [314.9, 511.9]	333.5 [265.2, 421.2]	286.8 [229, 376.1]	216.3 [158.9, 291.4]
Calcium-to-magnesium intake ratio	1.60 [1.27, 1.96]	1.73 [1.42, 2.09]	1.74 [1.40, 2.15]	1.51 [1.15, 2.00]
Urinary albumin-to-creatinine ratio, mg/g	11 [6, 24]	12 [6.3, 24]	12 [6.2, 26]	11.7 [6, 27]
Age, years	69.4 ± 8.9	69.0 ± 9.4	69.2 ± 10.0	67.6 ± 11.1
Male sex, n (%)	654 (38.2)	706 (41.2)	873 (51.0)	1,101 (64.3)
Body mass index, kg/m^2^	22.7 ± 3.0	22.6 ± 3.1	22.7 ± 3.1	22.7 ± 3.1
eGFR, mL/min/1.73 m^2^	73.9 ± 14.9	73.7 ± 14.7	74.7 ± 16.0	74.9 ± 17.3
Current smoker, n (%)	198 (11.6)	168 (9.8)	218 (12.7)	369 (21.5)
Never or rarely drinking, n (%)	975 (57.0)	890 (52.0)	777 (45.4)	617 (36.0)
Regular exercise habit, n (%)	701 (41.0)	666 (38.9)	621 (36.3)	544 (31.8)
Diabetes, n (%)	183 (10.7)	141 (8.2)	184 (10.8)	168 (9.8)
Hypertension, n (%)	815 (47.6)	869 (50.7)	915 (53.5)	947 (55.3)
History of urinary tract stone, n (%)	64 (3.7)	75 (4.4)	79 (4.6)	101 (5.9)

*Note.* Values are presented as the mean ± standard deviation, median (interquartile interval), or number (percentage). ^a^Nutrients were adjusted by energy intake using the residual method. eGFR, estimated glomerular filtration rate.

Spearman’s correlation coefficient between calcium and magnesium intake was 0.551. The calcium-to-magnesium intake ratio showed a stronger correlation with calcium intake than with magnesium intake (Spearman’s correlation coefficients: 0.755 for calcium and 0.035 for magnesium). The urinary calcium-to-magnesium ratio showed a stronger inverse correlation with magnesium intake than with calcium intake (Spearman’s correlation coefficients: −0.042 for calcium and −0.070 for magnesium). The LOWESS curves showed an inverse relationship of calcium intake and the calcium-to-magnesium intake ratio with albuminuria but a slight J-shaped relationship of magnesium intake with albuminuria (S1 Fig.).

In multivariable linear regression analysis, calcium and magnesium intakes were inversely associated with albuminuria (Models 1 and 2) but, after mutual adjustment, only calcium intake was independently associated with albuminuria (regression coefficient [95%CI] in Model 3, −0.084 [−0.149, −0.019]) (Table 2). The calcium-to-magnesium intake ratio was inversely associated with albuminuria (regression coefficient [95%CI] in Model 2, −0.085 [−0.150, −0.021]). Overall, these results were significant in the total sample and in male participants.

**Table 2:** Multivariable linear regression analysis of natural logarithms of calcium and magnesium intakes and the calcium-to-magnesium intake ratio with natural logarithms of the urinary albumin-to-creatinine ratio.

	Total		Men		Women	
	β (95% CI)	Standardized β	β (95% CI)	Standardized β	β (95% CI)	Standardized β
**Dietary calcium intake, mg/day**						
Model 1	−0.133 (−0.189, −0.077)	−0.057	−0.142 (−0.223, −0.062)	−0.060	−0.116 (−0.194, −0.038)	−0.047
Model 2	−0.110 (−0.164, −0.057)	−0.047	−0.138 (−0.215, −0.060)	−0.058	−0.089 (−0.163, −0.014)	−0.036
Model 3	−0.084 (−0.149, −0.019)	−0.036	−0.116 (−0.211, −0.020)	−0.049	−0.06 (−0.149, 0.029)	−0.024
**Dietary magnesium intake, mg/day**						
Model 1	−0.220 (−0.322, −0.118)	−0.050	−0.24 (−0.389, −0.090)	−0.054	−0.190 (−0.330, −0.049)	−0.042
Model 2	−0.172 (−0.270, −0.074)	−0.039	−0.195 (−0.339, −0.051)	−0.044	−0.153 (−0.288, −0.018)	−0.034
Model 3	−0.085 (−0.204, 0.035)	−0.019	−0.070 (−0.247, 0.107)	−0.016	−0.094 (−0.254, 0.066)	−0.021
**Calcium-to-magnesium intake ratio**						
Model 1	−0.099 (−0.166, −0.032)	−0.036	−0.116 (−0.214, −0.018)	−0.041	−0.078 (−0.169, 0.012)	−0.029
Model 2	−0.085 (−0.150, −0.021)	−0.031	−0.124 (−0.218, −0.030)	−0.044	−0.056 (−0.143, 0.031)	−0.021

*Note.* Dietary calcium and magnesium intakes were adjusted for energy intake by the residual method. Calcium and magnesium intakes and the urinary albumin-to-creatinine ratio were converted to the natural logarithm in the multivariable linear regression model. Model 1 was adjusted for age, survey area, current smoker, never or rarely drinking, regular exercise habit, and energy intake (quartile). Model 2 was further adjusted for body mass index, hypertension, diabetes, history of urinary tract stone, and estimated glomerular filtration rate. Model 3 was further mutually adjusted for magnesium and calcium intakes.

The results in multivariable logistic regression analysis for microalbuminuria were largely similar in Models 1 and 2, but neither calcium nor magnesium intakes showed statistical significance in Model 3 (S3 Table). Lower calcium-to-magnesium intake ratio was associated with a higher odds ratio of microalbuminuria (*P* for trend = 0.028 in Model 2). In the sex-stratified analysis, this association was significant in men but not in women. When we performed multivariable logistic regression analysis for high normoalbuminuria, we found that calcium intake and calcium-to-magnesium intake ratio were not significantly associated with high normoalbuminuria in any adjusted models (S4 Table). Magnesium intake had a higher odds ratio for high normoalbuminuria in all three models (top vs bottom quartile, adjusted odds ratio [95% CI], 1.24 [1.04, 1.48], *P* for trend = 0.032 in Model 3). In the sex-stratified analysis, this association was significant in men but not in women.

**Fig 1:**
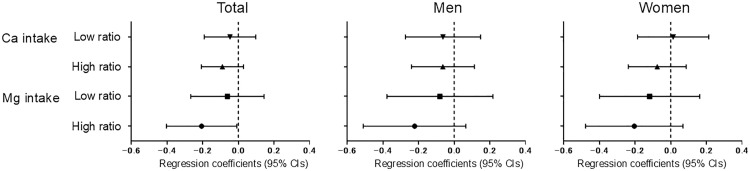
Associations of calcium and magnesium intakes with albuminuria in participants divided by the level of the calcium-to-magnesium intake ratio. *Note.* Dietary calcium and magnesium intakes were adjusted for energy intake by the residual method. In the multivariable linear regression model, calcium and magnesium intakes and the urinary albumin-to-creatinine ratio were converted to the natural logarithm. The participants were divided by median value of dietary calcium-to-magnesium ratio at 1.65, 1.53, and 1.75 for total, men, and women, respectively. The models were adjusted by age, sex, survey area, current smoker, never or rarely drinking, regular exercise habit, energy intake (quartile), diabetes, hypertension, history of urinary tract stone, body mass index, eGFR, and mutual magnesium or calcium intake. Ca, calcium; CI, confidence interval; eGFR, estimated glomerular filtration rate; Mg, magnesium.

Next, we performed a multivariable linear regression analysis of calcium and magnesium intakes with albuminuria according to groups divided by intake ratio level (Fig 1). The association of calcium and magnesium intakes with albuminuria was consistent across the intake ratio group overall (all *P* for interaction > 0.05). Although it did not reach statistical significance (*P* for interaction = 0.109), the more evident inverse association between magnesium intake and albuminuria was seen in participants with a high intake ratio compared with those with a low intake ratio.

**Fig 2:**
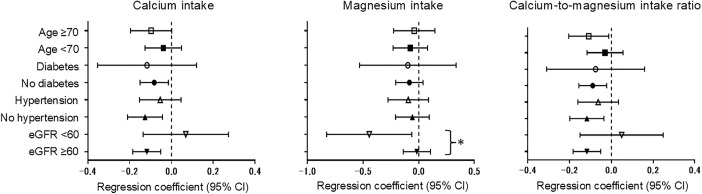
Regression coefficients (95% CIs) of the natural logarithms of calcium and magnesium intakes and intake ratio for the natural logarithm of the urinary albumin-to-creatinine ratio by subgroup. *Note.* Dietary calcium and magnesium intakes were adjusted for energy intake by the residual method. Calcium and magnesium intakes and the urinary albumin-to-creatinine ratio were converted to the natural logarithm in the multivariable linear regression model. Regression coefficients (95% CI) were adjusted for age, sex, survey area, current smoker, never or rarely drinking, regular exercise habit, energy intake (quartile), body mass index, hypertension, diabetes, history of urinary tract stone, estimated glomerular filtration rate and mutually adjusted for magnesium and calcium intakes (except for the model for intake ratio). The variable defining a relevant subgroup was not included in that subgroup analysis model (e.g., age was not included in the model when we analyzed age subgroups). * indicates *P* for interaction <0.05. CI, confidence interval; eGFR, estimated glomerular filtration rate.

In a subgroup analysis, we observed inverse relationships between calcium and magnesium intakes and intake ratio with albuminuria in most subgroups (Fig 2). A significant interaction was seen only in the kidney function group for magnesium intake (*P* for interaction = 0.012). In participants with eGFR < 60 mL/1.73 m^2^, the inverse association between magnesium intake and albuminuria was more evident than in those with eGFR > 60 mL/min/1.73 m^2^.

We repeated the linear and logistic regression analyses after the exclusion of participants who used calcium or magnesium supplements (N = 6,502, S5 and S6 Tables). The results were generally consistent with those in the entire analytic population.

Finally, we performed similar analyses using the urinary calcium-to-magnesium ratio instead of the intake ratio (N = 6,282). In multivariable linear regression analysis, a significant inverse association between the natural log-transformed calcium-to-magnesium intake ratio and the natural log-transformed ACR was observed in all participants and in male participants (S7 Table). In multivariable logistic regression analysis, significant associations of the urinary calcium-to-magnesium ratio with microalbuminuria were seen in Model 1 in men and women but this was attenuated by clinical factors in Model 2 (S8 Table). These associations exhibited opposing directions between men and women (*P* for sex interaction <0.05).

## Discussion

In this cross-sectional study, we observed that calcium and magnesium intakes were inversely associated with albuminuria but, after mutual adjustment, only calcium intake remained significantly associated with albuminuria. The dietary and urinary calcium-to-magnesium intake ratios were inversely associated with albuminuria. These associations were evident overall and in male participants. We observed a significant inverse association between magnesium intake and high normoalbuminuria; however, this was not the case for calcium intake or the calcium-to-magnesium intake ratio. When stratified by intake ratio levels, we did not observe a significant interaction for the association of calcium and magnesium intake with albuminuria. However, the strength of the association between magnesium intake and albuminuria appeared to differ across intake ratio groups; participants with a higher intake ratio showed a stronger inverse association than those with a lower intake ratio. Overall, the associations of calcium intake and intake ratio with albuminuria were robust and consistent across different groups.

This study found that lower calcium intake was associated with albuminuria independent of magnesium intake. To our knowledge, no studies have examined calcium intake and kidney variables other than kidney stones in the general population. Considering that albuminuria is a strong risk factor for adverse cardiovascular outcomes [3], the possible link between calcium intake and cardiovascular mortality would be worth discussing. Although our findings are generally linear, some previous studies showed a U-shaped relationship between calcium intake and cardiovascular mortality [18,19]. This inconsistency may be due to different outcomes and study design. Moreover, caution regarding differences in calcium intake levels across study populations is warranted when discussing inconsistency. Our study population had lower calcium intake levels compared with those in the Western countries [20] and previous studies have suggested that there was a threshold effect of calcium intake for health outcomes [18,19].

In some previous studies, magnesium intake was inversely associated with CKD in the general population but was not inversely associated with albuminuria in the present study after accounting for calcium intake. Previous research found an inverse dose–response relationship between dietary magnesium intake and incident CKD but did not account for calcium intake [21,22]. One cross-sectional study showed a non-linear relationship between magnesium intake and CKD prevalence independent of calcium intake levels among adults aged 50 years or older, based on data from the National Health and Nutrition Examination Survey (NHANES) 2007–2018 [23]. A possible reason for this inconsistency is that, as noted previously, although magnesium intake levels are similar (326.1 mg in the present study [energy-adjusted median] vs 322.3 mg in the NHANES study [mean]), calcium intake levels differ across study populations (567.9 mg vs 853.0 mg). The present study also found that lower magnesium intake was associated with a higher odds ratio of high normoalbuminuria independent of calcium intake levels. This might reflect the remaining benefits of magnesium intake at the pre-disease state of albuminuria.

Notably, we observed a significant association of low calcium-to-magnesium intake ratio with albuminuria. Previous studies have examined serum calcium-to-magnesium ratio and kidney measures but did not assess dietary intake ratio. They tested the association of serum calcium-to-magnesium ratio with total/cardiovascular mortality in hemodialysis patients and with initiation of renal replacement therapy in predialysis CKD patients (eGFR 10–30 mL/min/1.73 m^2^) [24,25]. Although the findings of these previous studies were not in line with our results, it is difficult to compare them with our study because of differences in the study populations (hemodialysis/predialysis CKD patients vs community-dwelling adults). Also, we should acknowledge that blood concentrations of calcium and magnesium did not always reflect dietary intake due to limited representativeness of the total body store (about <1%) and because their levels are maintained within a narrow homeostatic range [1,26].

Although the interaction was not statistically significant in the present study, a significant inverse association between magnesium intake and albuminuria was found in participants with a high intake ratio, as in a prior study that tested the association with mortality [10]. Analysis of data from the NHANES 2001–2011 population with a high intake ratio (above 2.60) revealed that a high calcium intake with inadequate magnesium intake was associated with high odds of metabolic syndrome [27]. We are unsure of the underlying mechanisms for these observations; however, considering magnesium as a calcium antagonist [28], we should consider intake ratio when examining the association between magnesium intake and health outcomes, especially in populations with high intake ratio.

We cannot fully explain the mechanism of the inverse association of calcium intake and the calcium-to-magnesium intake ratio with albuminuria; however, our observations may indicate the existence of shared pathophysiological segments of the kidneys related to the reabsorption of filtered calcium, magnesium, and albumin. Glomerular-filtered albumin is reabsorbed mainly by the proximal tubule (about 70%) [29] and a large amount of filtered calcium is also reabsorbed via the proximal tubule [30]. Meanwhile, magnesium reabsorption in the proximal tubule is not altered by dietary magnesium intake, whereas the distal tubule matches reabsorption from magnesium intake [31]. Therefore, calcium intake may be more strongly associated with albuminuria than magnesium intake.

Moreover, calcium and magnesium intake may affect chronic inflammation and oxidative stress, conditions that are associated with albuminuria as well [32,33]. To our knowledge, previous studies have largely focused on the associations between those conditions and magnesium intake [34]. Further research is needed to elucidate the effects of calcium intake on chronic inflammation and oxidative stress.

We observed an interaction by kidney function in the association between magnesium intake and albuminuria. The inverse association between magnesium intake and albuminuria was more evident in participants with eGFR < 60 mL/min/1.73 m^2^ than in those with eGFR ≥ 60 mL/min/1.73 m^2^. In patients with CKD, intake of phosphorous can elevate its serum levels, leading to an increase in urinary protein excretion, although magnesium intake may be able to attenuate such phosphorus effects [35]. In addition, patients with CKD are known to have elevated serum magnesium levels alongside increasing CKD severity [36]. Because hypomagnesemia was associated with faster decline in kidney function compared with elevated magnesium levels in patients with non-diabetic CKD [37], elevated serum magnesium—possibly derived from dietary magnesium intake—might have had a favorable impact on albuminuria. However, we are not certain of an exact reason for this interaction in relation to albuminuira, further studies are needed to confirm our observation.

Interestingly, sex modified the association of the dietary and urinary calcium-to-magnesium ratios with microalbuminuria. Female participants demonstrated the opposite direction or neutral of the association with albuminuria compared with male participants. Renal calcium and magnesium handling in the distal convoluted tubule is regulated by estrogen [38,39]. Estrogen receptors also play a protective role in megalin-mediated protein reabsorption, which participates in albumin handling in the proximal convoluted tubule [40]. Given that most of the female participants in the present study were postmenopausal, their reduced calcium absorption efficiency through the intestine, due to decreased estrogen levels [41], might have attenuated the health impact of dietary calcium. These facts may support our observation that sex could alter the association of calcium and magnesium balance with albuminuria in the kidneys.

This study has a few implications. Based on our results, calcium intake and calcium-to-magnesium intake ratio are potential modifiable factors for preventing albuminuria. Given the limited information on dietary modifiable factors for preventing CKD, specifically albuminuria, our results provide novel insights into kidney health. Simultaneously, we should acknowledge that the significant inverse association between magnesium intake and albuminuria was seen in specific groups, including participants with a high intake ratio and eGFR < 60 mL/min/1.73 m^2^. Regarding the high intake ratio group, given that previous studies have suggested that there is a threshold for the health effects of calcium intake [18,19], magnesium is important to maintain an appropriate balance with calcium. Regarding the eGFR < 60 mL/min/1.73 m^2^ group, given the favorable relationship between a plant-based diet (main sources of dietary magnesium) and CKD [42,43], our findings further support the current evidence on the association between a plant-based diet and kidney health.

Several limitations should be noted. First, this study measured ACR only once in the spot urine sample. This might cause a misclassification of albuminuria and a misinterpretation of its chronicity. Second, although our FFQ has been validated, the correlation coefficients of calcium and magnesium between the FFQ and a 12-day weighted food record showed a moderate correlation and those of the ratio were not reported [15]. Self-reported FFQ raised concerns about recall bias and inaccurate responses regarding calcium and magnesium intake levels. This bias might attenuate the estimates associated with albuminuria toward null. Moreover, we could not assess the intakes of calcium and magnesium from drinking water. Based on a Japanese national report, the average intakes of calcium and magnesium from water were 15.6 mg/day (2.9% of the total intake) and 13.3 mg/day (5.1%), respectively, suggesting that water contributes little to the total intake [44]. Third, estimations of calcium and magnesium intakes based on self-reported FFQ might not perfectly reflect the actual amount absorbed, utilized, and subsequently excreted by the body. For magnesium in particular, various factors, including kidney function and gastrointestinal conditions (e.g., diarrhea), can influence urinary excretion. In addition, we were unable to consider medications that might affect the absorption and excretion of calcium and magnesium such as proton pump inhibitors and diuretics. We did not obtain full information on vitamin D_3_ supplementation use, which can significantly affect calcium absorption; this should be acknowledged as a notable limitation of the study. Fourth, as mentioned previously, the generalizability of our findings should be carefully considered because our population was recruited from only one area in Japan. Finally, this was a cross-sectional observational study, and thus causality and temporality between calcium and magnesium intakes and albuminuria could not be determined. Nonetheless, this study had some strengths, including its holistic assessment of the associations between calcium and magnesium intake and albuminuria, as well as its careful evaluation of modification effects across key groups.

## Conclusions

Although magnesium intake was associated with albuminuria only before accounting for calcium intake, calcium intake and the calcium-to-magnesium intake ratio were inversely associated with albuminuria in middle-aged and older Japanese adults. The association of calcium or magnesium intake with albuminuria was not modified by the intake ratio; however, when stratified by the intake ratio level, magnesium intake was inversely associated with albuminuria in participants with a high intake ratio. Our findings extend the significance of these minerals and their balance to kidney health, especially albuminuria. Future studies are needed to confirm our observations in other populations with different dietary habits and to verify the longitudinal associations of calcium and magnesium intakes and their intake ratio with albuminuria development and progression.

## Supporting information

S1 Fig Locally weighted scatter plot smoothing curves of calcium and magnesium intakes and the calcium-to-magnesium intake ratio with albuminuria. (TIF)

S1 Table Descriptive characteristics according to quartiles of the calcium-to-magnesium intake ratio. (PDF)

S2 Table Prevalence of microalbuminuria according to quartiles of calcium and magnesium intake and their ratio. (PDF)

S3 Table Adjusted odds ratio (95% CIs) of microalbuminuria for the quartiles of dietary intakes of calcium and magnesium and the calcium-to-magnesium intake ratio. (PDF)

S4 Table Adjusted odds ratio (95% CIs) of high normoalbuminuria for the quartiles of dietary intakes of calcium and magnesium and the calcium-to-magnesium intake ratio. (PDF)

S5 Table Multivariable linear regression analysis of natural logarithms of calcium and magnesium intakes and the calcium-to-magnesium intake ratio with natural logarithm of the urinary albumin-to-creatinine ratio in the participants after excluding calcium or magnesium supplement users. (PDF)

S6 Table Adjusted odds ratio (95% CIs) of microalbuminuria for the quartiles of dietary intakes of calcium and magnesium and the calcium-to-magnesium intake ratio in the participants after excluding calcium or magnesium supplement users. (PDF)

S7 Table Multivariable linear regression analysis between the natural logarithms of the urinary calcium-to-magnesium ratio and albumin-to-creatinine ratio. (PDF)

S8 Table Adjusted odds ratio (95% CIs) of microalbuminuria for the quartiles of the urinary calcium-to-magnesium ratio. (PDF)
