# Supplementary figures and images for: Associations of calcium and magnesium intakes and their intake ratio with albuminuria in middle-aged and older adults
Source: PLoS One. 2025 Nov 26;20(11):e0335412. doi: 10.1371/journal.pone.0335412 (PMC12654892; doi:10.1371/journal.pone.0335412)

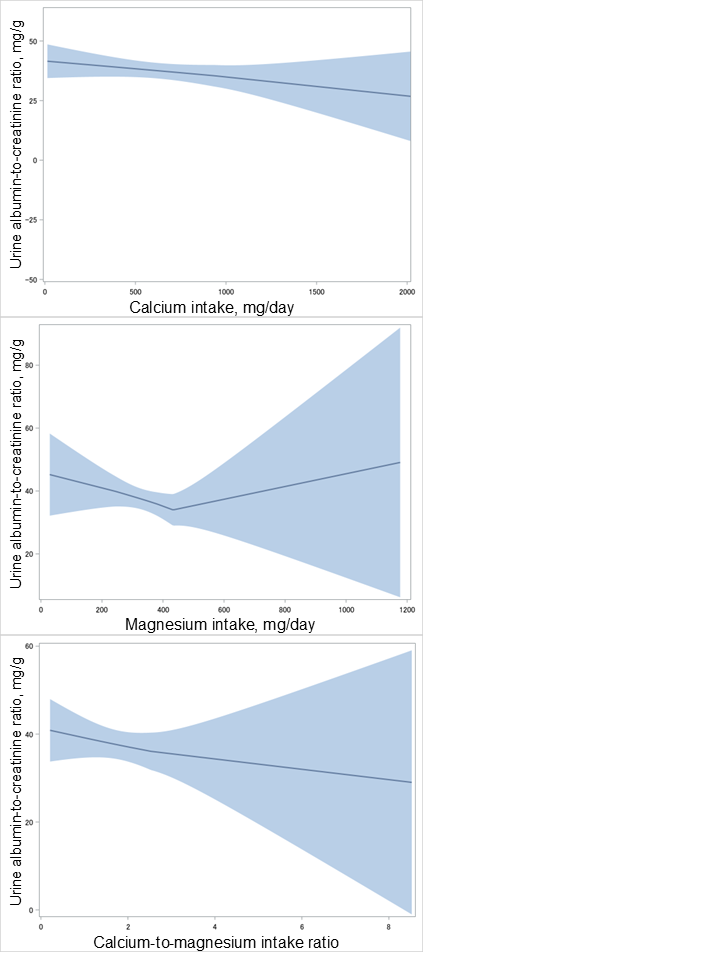

Supplement: S1 Fig — (TIF) [file pone.0335412.s001.tif]
