# Supplementary material for: Associations of calcium and magnesium intakes and their intake ratio with albuminuria in middle-aged and older adults
Source: PLoS One. 2025 Nov 26;20(11):e0335412. doi: 10.1371/journal.pone.0335412 (PMC12654892; doi:10.1371/journal.pone.0335412)
Supplement: S1 Table — (PDF) [file pone.0335412.s002.pdf]

S1 Table. Descriptive characteristics according to quartiles of the calcium-to-magnesium intake ratio

	Quartile of calcium-to-magnesium intake ratio			
	≥2.06	1.65–2.05	1.30–1.64	<1.30
N	1,713	1,711	1,712	1,713
Energy-unadjusted calcium, mg/day	831.2 [579.7, 1415.6]	605.4 [465.7, 774.9]	463.6 [347.8, 613.1]	270.9 [178.4, 382.6]
Energy-unadjusted magnesium, mg/day	333.1 [239.8, 452.4]	331 [254.7, 421.3]	316.4 [236.9, 407.8]	256.9 [180.5, 352.5]
Dietary calcium-to-magnesium ratio	2.53 [2.23, 3.28]	1.83 [1.74, 1.94]	1.48 [1.4, 1.57]	1.09 [0.93, 1.20]
Urinary albumin-to-creatinine ratio, mg/g	12 [7, 26]	12 [6.7, 24]	11 [6, 25]	11 [6, 26]
Age, years	70.6 ± 9.8	69.6 ± 9.3	68.3 ± 9.8	66.8 ± 10.4
Male sex, n (%)	675 (39.4)	726 (42.4)	839 (49.0)	1,094 (63.9)
Body mass index, kg/m^2	22.6 ± 3.0	22.7 ± 3.1	22.6 ± 3.1	22.7 ± 3.0
eGFR, mL/min/1.73 m^2	73.0 ± 15.6	73.6 ± 15.7	74.8 ± 15.5	75.9 ± 16.1
Current smoker, n (%)	151 (8.8)	163 (9.5)	264 (15.4)	375 (21.9)
Never or rarely drinking, n (%)	967 (56.5)	885 (51.7)	783 (45.7)	624 (36.4)
Regular exercise habit, n (%)	675 (39.4)	701 (41.0)	599 (35.0)	557 (32.5)
Diabetes, n (%)	179 (10.5)	149 (8.7)	169 (9.9)	179 (10.5)
Hypertension, n (%)	868 (50.7)	884 (51.7)	882 (51.5)	912 (53.2)
History of urinary tract stone, n (%)	71 (4.1)	79 (4.6)	82 (4.8)	87 (5.1)

Values are presented as the mean ± standard deviation, median (interquartile interval), or number (percentage). eGFR, estimated glomerular filtration rate.
