# Supplementary material for: Associations of calcium and magnesium intakes and their intake ratio with albuminuria in middle-aged and older adults
Source: PLoS One. 2025 Nov 26;20(11):e0335412. doi: 10.1371/journal.pone.0335412 (PMC12654892; doi:10.1371/journal.pone.0335412)
Supplement: S2 Table — (PDF) [file pone.0335412.s003.pdf]

S2 Table. Prevalence of microalbuminuria according to quartiles of calcium and magnesium intake and their ratio

	Prevalence of microalbuminuria (%)				
	Highest quartile	Q3	Q2	Lowest quartile	*P*-value
Calcium intake	20.6	21.6	22.6	20.9	0.491
Magnesium intake	20.4	19.9	22.6	22.8	0.080
Calcium-to-magnesium intake ratio	21.6	20.7	21.8	21.6	0.863

Microalbuminuria was defined as a urinary albumin-to-creatinine ratio of 30 mg/g or more. *P*-values are obtained by the chi-squared test.
