# Supplementary material for: Associations of calcium and magnesium intakes and their intake ratio with albuminuria in middle-aged and older adults
Source: PLoS One. 2025 Nov 26;20(11):e0335412. doi: 10.1371/journal.pone.0335412 (PMC12654892; doi:10.1371/journal.pone.0335412)
Supplement: S3 Table — (PDF) [file pone.0335412.s004.pdf]

S3 Table. Adjusted odds ratio (95% CIs) of microalbuminuria for the quartiles of dietary intakes of calcium and magnesium and the calcium-to-magnesium intake ratio

	Total				*P* for trend	Men				*P* for trend	Women				*P* for trend
**Dietary calcium intake, mg/day**															
Range	≥746.4	567.9–746.3	419.7–567.8	<419.7		≥667.7	492.5–667.6	492.4–359.6	<359.6		≥805.0	633.1–805.0	487.9–633.0	<487.9	
Cases, n (%)	353 (20.6)	370 (21.6)	387 (22.6)	357 (20.9)		169 (20.3)	183 (21.9)	175 (21.0)	170 (20.4)		194 (22.1)	188 (21.4)	187 (21.3)	201 (22.9)	
Model 1	Reference	1.14 [0.96, 1.35]	1.31 [1.11, 1.56]	1.29 [1.08, 1.54]	0.002	Reference	1.19 [0.94, 1.52]	1.25 [0.98, 1.60]	1.29 [1.001, 1.67]	0.046	Reference	1.04 [0.82, 1.31]	1.09 [0.86, 1.39]	1.24 [0.98, 1.57]	0.071
Model 2	Reference	1.12 [0.94, 1.34]	1.29 [1.08, 1.54]	1.22 [1.01, 1.47]	0.013	Reference	1.12 [0.87, 1.43]	1.22 [0.95, 1.57]	1.28 [0.98, 1.66]	0.053	Reference	1.01 [0.79, 1.28]	1.06 [0.83, 1.35]	1.16 [0.91, 1.48]	0.210
Model 3	Reference	1.12 [0.94, 1.33]	1.25 [1.04, 1.50]	1.12 [0.91, 1.38]	0.126	Reference	1.10 [0.85, 1.41]	1.16 [0.89, 1.51]	1.14 [0.84, 1.55]	0.315	Reference	1.00 [0.78, 1.28]	1.04 [0.81, 1.34]	1.09 [0.83, 1.43]	0.462
**Dietary magnesium intake, mg/day**															
Range	≥381.1	326.1–381.0	279.8–326.0	<279.8		≥364.4	309.9–364.3	262.3–309.8	<262.3		≥395.7	341.5–395.6	296.3–341.4	<296.3	

Cases, n (%)	349 (20.4)	341 (19.9)	386 (22.6)	391 (22.8)		165 (19.8)	170 (20.4)	169 (20.3)	193 (23.2)		177 (20.1)	190 (21.6)	190 (21.6)	213 (24.2)	
Model 1	Reference	0.99 [0.84, 1.18]	1.15 [0.97, 1.37]	1.25 [1.05, 1.49]	0.003	Reference	1.08 [0.84, 1.38]	1.11 [0.87, 1.43]	1.35 [1.05, 1.72]	0.019	Reference	1.08 [0.85, 1.37]	1.01 [0.80, 1.29]	1.23 [0.97, 1.56]	0.140
Model 2	Reference	0.98 [0.82, 1.17]	1.12 [0.94, 1.33]	1.21 [1.01, 1.44]	0.017	Reference	1.09 [0.85, 1.40]	1.14 [0.89, 1.48]	1.31 [1.02, 1.69]	0.035	Reference	1.07 [0.84, 1.37]	0.98 [0.77, 1.25]	1.22 [0.96, 1.55]	0.190
Model 3	Reference	0.95 [0.79, 1.14]	1.06 [0.89, 1.28]	1.15 [0.94, 1.41]	0.173	Reference	1.06 [0.82, 1.37]	1.10 [0.84, 1.43]	1.24 [0.92, 1.66]	0.186	Reference	1.06 [0.83, 1.36]	0.96 [0.74, 1.24]	1.17 [0.89, 1.54]	0.408
**Calcium-to-magnesium intake ratio**															
Range	≥2.06	1.65–2.05	1.30–1.64	<1.30		≥1.94	1.53–1.93	1.19–1.52	<1.19		≥2.15	1.75–2.14	1.43–1.74	<1.43	
Cases, n (%)	370 (21.6)	354 (20.7)	373 (21.8)	370 (21.6)		161 (19.3)	169 (20.3)	188 (22.6)	179 (21.5)		198 (22.5)	199 (22.6)	183 (20.8)	190 (21.6)	
Model 1	Reference	1.03 [0.87, 1.22]	1.17 [0.99, 1.39]	1.23 [1.03, 1.47]	0.010	Reference	1.14 [0.89, 1.46]	1.40 [1.10, 1.79]	1.31 [1.02, 1.70]	0.012	Reference	1.13 [0.89, 1.43]	1.07 [0.84, 1.36]	1.17 [0.91, 1.49]	0.306
Model 2	Reference	1.03 [0.87, 1.23]	1.15 [0.97, 1.37]	1.20 [0.998, 1.44]	0.028	Reference	1.16 [0.90, 1.50]	1.41 [1.09, 1.81]	1.36 [1.05, 1.78]	0.008	Reference	1.10 [0.87, 1.40]	1.06 [0.83, 1.36]	1.08 [0.84, 1.39]	0.649

Intakes of calcium and magnesium were adjusted by energy intake using the residual method. Intakes of calcium and magnesium and the intake ratio were divided by each quartile group. Microalbuminuria was defined as a urinary albumin-to-creatinine ratio of 30 mg/g or more. Model 1 was adjusted for age, sex, survey area, current smoker, never or rarely drinking, regular exercise habit, and energy intake (quartile). Model 2 was further adjusted for body mass index, hypertension, diabetes, history of urinary tract stone, and estimated glomerular filtration rate. As for intakes of calcium and magnesium, Model 3 was further mutually adjusted for the intakes of calcium and magnesium.
