# Supplementary material for: Associations of calcium and magnesium intakes and their intake ratio with albuminuria in middle-aged and older adults
Source: PLoS One. 2025 Nov 26;20(11):e0335412. doi: 10.1371/journal.pone.0335412 (PMC12654892; doi:10.1371/journal.pone.0335412)
Supplement: S4 Table — (PDF) [file pone.0335412.s005.pdf]

S4 Table. Adjusted odds ratio (95% CIs) of high normoalbuminuria for the quartiles of dietary intakes of calcium and magnesium and the calcium-to-magnesium intake ratio

	Total				*P* for trend	Men				*P* for trend	Women				*P* for trend
**Dietary calcium intake, mg/day**															
Range	≥746.4	567.9–746.3	419.7–567.8	<419.7		≥667.7	492.5–667.6	492.4–359.6	<359.6		≥805.0	633.1–805.0	487.9–633.0	<487.9	
Cases, n (%)	1,013 (59.2)	1,032 (60.3)	965 (56.3)	911 (53.2)		438 (52.6)	439 (52.6)	399 (47.9)	416 (49.9)		560 (63.8)	562 (63.9)	561 (63.8)	546 (62.2)	
Model 1	Reference	1.12 [0.97, 1.29]	1.10 [0.95, 1.27]	1.17 [0.999, 1.36]	0.075	Reference	1.06 [0.87, 1.30]	0.97 [0.79, 1.19]	1.14 [0.92, 1.4]	0.392	Reference	1.08 [0.88, 1.32]	1.16 [0.95, 1.43]	1.16 [0.94, 1.43]	0.127
Model 2	Reference	1.12 [0.97, 1.3]	1.07 [0.93, 1.25]	1.10 [0.94, 1.28]	0.354	Reference	1.02 [0.83, 1.26]	0.94 [0.76, 1.16]	1.10 [0.89, 1.37]	0.573	Reference	1.08 [0.88, 1.34]	1.15 [0.93, 1.42]	1.10 [0.89, 1.37]	0.329
Model 3	Reference	1.10 [0.95, 1.28]	1.03 [0.88, 1.2]	0.99 [0.83, 1.19]	0.865	Reference	0.995 [0.81, 1.23]	0.87 [0.70, 1.09]	0.94 [0.74, 1.21]	0.454	Reference	1.08 [0.87, 1.33]	1.13 [0.91, 1.40]	1.05 [0.82, 1.33]	0.588
**Dietary magnesium intake, mg/day**															
Range	≥381.1	326.1–381.0	279.8–326.0	<279.8		≥364.4	309.9–364.3	262.3–309.8	<262.3		≥395.7	341.5–395.6	296.3–341.4	<296.3	

Cases, n (%)	966 (56.4)	1,000 (58.4)	989 (57.8)	966 (56.4)		407 (48.9)	423 (50.7)	415 (49.8)	447 (53.7)		543 (61.8)	552 (62.9)	568 (64.6)	566 (64.4)	
Model 1	Reference	1.13 [0.98, 1.31]	1.15 [0.998, 1.33]	1.26 [1.09, 1.46]	0.003	Reference	1.10 [0.90, 1.35]	1.10 [0.90, 1.35]	1.35 [1.1, 1.66]	0.006	Reference	1.03 [0.84, 1.26]	1.08 [0.88, 1.33]	1.16 [0.94, 1.43]	0.136
Model 2	Reference	1.14 [0.98, 1.32]	1.12 [0.96, 1.3]	1.22 [1.05, 1.42]	0.02	Reference	1.10 [0.90, 1.36]	1.12 [0.91, 1.38]	1.31 [1.06, 1.62]	0.015	Reference	1.03 [0.83, 1.27]	1.08 [0.87, 1.33]	1.12 [0.91, 1.39]	0.256
Model 3	Reference	1.13 [0.97, 1.31]	1.12 [0.96, 1.31]	1.24 [1.04, 1.48]	0.032	Reference	1.13 [0.91, 1.39]	1.16 [0.93, 1.44]	1.37 [1.07, 1.74]	0.013	Reference	1.01 [0.81, 1.25]	1.06 [0.85, 1.32]	1.11 [0.87, 1.40]	0.427
**Calcium-to-magnesium intake ratio**															
Range	≥2.06	1.65–2.05	1.30–1.64	<1.30		≥1.94	1.53–1.93	1.19–1.52	<1.19		≥2.15	1.75–2.14	1.43–1.74	<1.43	
Cases, n (%)	1,028 (60.0)	999 (58.4)	951 (55.6)	943 (55.1)		435 (52.2)	423 (50.8)	408 (49.0)	426 (51.1)		573 (65.2)	549 (62.5)	560 (63.8)	547 (62.2)	
Model 1	Reference	1.00 [0.87, 1.16]	0.99 [0.85, 1.14]	1.12 [0.96, 1.30]	0.215	Reference	0.98 [0.80, 1.19]	1.02 [0.83, 1.25]	1.12 [0.91, 1.38]	0.279	Reference	0.95 [0.77, 1.17]	1.07 [0.86, 1.32]	1.06 [0.85, 1.31]	0.419
Model 2	Reference	0.99 [0.86, 1.15]	0.96 [0.83, 1.12]	1.08 [0.92, 1.26]	0.496	Reference	0.97 [0.79, 1.19]	0.99 [0.81, 1.23]	1.11 [0.89, 1.37]	0.371	Reference	0.92 [0.74, 1.14]	1.05 [0.85, 1.3]	0.98 [0.79, 1.23]	0.809

Intakes of calcium and magnesium were adjusted by energy intake using the residual method. Intakes of calcium and magnesium and the intake ratio were divided by each quartile group. High normoalbuminuria was defined as a urinary albumin-to-creatinine ratio of 10 mg/g or more. Model 1 was adjusted for age, sex, survey area, current smoker, never or rarely drinking, regular exercise habit, and energy intake (quartile). Model 2 was further adjusted for body mass index, hypertension, diabetes, history of urinary tract stone, and estimated glomerular filtration rate. As for intakes of calcium and magnesium, Model 3 was further mutually adjusted for the intakes of calcium and magnesium.
