# Supplementary material for: Associations of calcium and magnesium intakes and their intake ratio with albuminuria in middle-aged and older adults
Source: PLoS One. 2025 Nov 26;20(11):e0335412. doi: 10.1371/journal.pone.0335412 (PMC12654892; doi:10.1371/journal.pone.0335412)
Supplement: S5 Table — (PDF) [file pone.0335412.s006.pdf]

S5 Table. Multivariable linear regression analysis of natural logarithms of calcium and magnesium intakes and the calcium-to-magnesium intake ratio with natural logarithm of the urinary albumin-to-creatinine ratio in the participants after excluding calcium or magnesium supplement users

	Total		Men		Women	
	β (95% CI)	Standardized β	β (95% CI)	Standardized β	β (95% CI)	Standardized β
**Dietary calcium intake, mg/day**						
Model 1	− 0.136 (− 0.194, − 0.079)	−0.058	−0.154 (−0.237, −0.072)	−0.065	−0.111 (−0.191, −0.03)	−0.045
Model 2	−0.113 (−0.169, −0.058)	−0.048	−0.148 (−0.227, −0.069)	−0.062	−0.083 (−0.16, −0.006)	−0.033
Model 3	−0.082 (−0.149, −0.014)	−0.035	−0.126 (−0.224, −0.028)	−0.053	−0.043 (−0.135, 0.049)	−0.017
**Dietary magnesium intake, mg/day**						
Model 1	−0.23 (−0.335, −0.126)	−0.052	−0.249 (−0.402, −0.096)	−0.056	−0.206 (−0.35, −0.062)	−0.046
Model 2	−0.187 (−0.288, −0.086)	−0.042	−0.207 (−0.354, −0.059)	−0.046	−0.173 (−0.311, −0.034)	−0.039
Model 3	−0.102 (−0.224, 0.021)	−0.023	−0.070 (−0.251, 0.111)	−0.016	−0.130 (−0.296, 0.035)	−0.029
**Calcium-to-magnesium intake ratio**						
Model 1	−0.098 (−0.167, −0.029)	−0.036	−0.130 (−0.230, −0.029)	−0.046	−0.059 (−0.152, 0.034)	−0.022
Model 2	−0.082 (−0.148, −0.016)	−0.030	−0.135 (−0.232, −0.039)	−0.048	−0.035 (−0.125, 0.054)	−0.013

Dietary calcium and magnesium intakes were adjusted for energy intake by the residual method. Calcium and magnesium intakes and the urinary albumin-to-creatinine ratio were converted to the natural logarithm in the multivariable linear regression model. Model 1 was adjusted for age, survey area, current smoker, never or rarely drinking, regular exercise habit, and energy intake (quartiles). Model 2 was further adjusted for body mass index, hypertension, diabetes, history of urinary tract stone, and estimated glomerular filtration rate. Model 3 was further mutually adjusted for magnesium and calcium intakes.
