# Supplementary material for: Associations of calcium and magnesium intakes and their intake ratio with albuminuria in middle-aged and older adults
Source: PLoS One. 2025 Nov 26;20(11):e0335412. doi: 10.1371/journal.pone.0335412 (PMC12654892; doi:10.1371/journal.pone.0335412)
Supplement: S6 Table — (PDF) [file pone.0335412.s007.pdf]

S6 Table. Adjusted odds ratio (95% CIs) of microalbuminuria for the quartiles of dietary intakes of calcium and magnesium and the calcium-to-magnesium intake ratio in the participants after excluding calcium or magnesium supplement users

	Total				*P* for trend	Men				*P* for trend	Women				*P* for trend
**Dietary calcium intake, mg/day**															
Range	≥741.9	563.3–741.8	417.6–563.2	<417.6		≥664.5	491.0–664.4	490.9–357.2	<357.2		≥801.0	629.0–800.9	485.1–628.9	<485.1	
Cases, n (%)	332 (20.4)	359 (22.1)	361 (22.2)	343 (21.1)		159 (20)	176 (22.2)	170 (21.4)	164 (20.6)		182 (21.9)	177 (21.4)	177 (21.3)	190 (22.9)	
Model 1	Reference	1.19 [1.00, 1.41]	1.30 [1.09, 1.55]	1.32 [1.10, 1.59]	0.002	Reference	1.25 [0.97, 1.60]	1.32 [1.02, 1.70]	1.36 [1.05, 1.77]	0.018	Reference	1.03 [0.81, 1.31]	1.10 [0.86, 1.40]	1.24 [0.98, 1.58]	0.070
Model 2	Reference	1.16 [0.97, 1.39]	1.29 [1.07, 1.54]	1.25 [1.03, 1.51]	0.011	Reference	1.18 [0.91, 1.52]	1.29 [0.995, 1.68]	1.35 [1.03, 1.76]	0.023	Reference	1.03 [0.80, 1.32]	1.06 [0.83, 1.37]	1.18 [0.92, 1.51]	0.201
Model 3	Reference	1.15 [0.96, 1.37]	1.23 [1.02, 1.49]	1.11 [0.90, 1.38]	0.183	Reference	1.16 [0.89, 1.50]	1.23 [0.94, 1.62]	1.20 [0.88, 1.65]	0.177	Reference	1.01 [0.78, 1.3]	1.03 [0.8, 1.34]	1.08 [0.81, 1.43]	0.549
**Dietary magnesium intake, mg/day**															
Range	≥380.7	325.7–380.6	279.6–325.6	<279.6		≥364.2	309.9–364.1	262.2–309.8	<262.2		≥395.1	340.9–395.0	295.8–340.9	<295.8	
Cases, n (%)	324 (19.9)	327 (20.1)	366 (22.5)	378 (23.3)		159 (20.0)	162 (20.4)	162 (20.4)	186 (23.4)		162 (19.5)	183 (22.1)	178 (21.5)	203 (24.5)	

Model 1	Reference	1.03 [0.86, 1.23]	1.19 [0.996, 1.41]	1.32 [1.11, 1.57]	0.001	Reference	1.07 [0.83, 1.38]	1.12 [0.87, 1.44]	1.35 [1.06, 1.74]	0.018	Reference	1.15 [0.9, 1.47]	1.03 [0.81, 1.32]	1.30 [1.02, 1.66]	0.086
Model 2	Reference	1.02 [0.85, 1.23]	1.15 [0.96, 1.38]	1.27 [1.06, 1.53]	0.004	Reference	1.07 [0.83, 1.39]	1.15 [0.88, 1.49]	1.33 [1.03, 1.72]	0.028	Reference	1.16 [0.9, 1.49]	1.02 [0.79, 1.31]	1.30 [1.01, 1.67]	0.107
Model 3	Reference	0.99 [0.82, 1.19]	1.10 [0.91, 1.33]	1.22 [0.99, 1.50]	0.065	Reference	1.03 [0.79, 1.34]	1.08 [0.83, 1.42]	1.22 [0.90, 1.65]	0.223	Reference	1.15 [0.89, 1.49]	0.999 [0.77, 1.31]	1.25 [0.94, 1.66]	0.249
**Calcium-to-magnesium intake ratio**															
Range	≥2.04	1.64–2.03	1.29–1.63	<1.29		≥1.93	1.5–1.92	1.19–1.51	<1.19		≥2.13	1.74–2.12	1.42–1.73	<1.42	
Cases, n (%)	354 (21.8)	335 (20.6)	352 (21.7)	354 (21.8)		149 (18.7)	166 (20.9)	182 (22.9)	172 (21.6)		191 (23)	185 (22.3)	175 (21.1)	175 (21.1)	
Model 1	Reference	1.01 [0.85, 1.20]	1.15 [0.97, 1.37]	1.23 [1.02, 1.47]	0.012	Reference	1.25 [0.97, 1.61]	1.50 [1.16, 1.93]	1.40 [1.08, 1.83]	0.004	Reference	1.08 [0.85, 1.37]	1.04 [0.81, 1.32]	1.08 [0.84, 1.39]	0.622
Model 2	Reference	1.02 [0.86, 1.22]	1.13 [0.95, 1.35]	1.20 [0.99, 1.45]	0.036	Reference	1.28 [0.99, 1.66]	1.49 [1.14, 1.93]	1.45 [1.10, 1.90]	0.004	Reference	1.06 [0.83, 1.36]	1.03 [0.80, 1.33]	1.01 [0.78, 1.31]	0.996

Intakes of calcium and magnesium were adjusted by energy intake using the residual method. Intakes of calcium and magnesium and the intake ratio were divided by each quartile group. Microalbuminuria was defined as a urinary albumin-to-creatinine ratio of 30 mg/g or more. Model 1 was adjusted for age, sex, survey area, current smoker, never or rarely drinking, regular exercise habit, and energy intake (quartile). Model 2 was further adjusted for body mass index, hypertension, diabetes, history of urinary tract stone, and estimated glomerular filtration rate. As for intakes of calcium and magnesium, Model 3 was further mutually adjusted for magnesium and calcium intakes.
