# Supplementary material for: Associations of calcium and magnesium intakes and their intake ratio with albuminuria in middle-aged and older adults
Source: PLoS One. 2025 Nov 26;20(11):e0335412. doi: 10.1371/journal.pone.0335412 (PMC12654892; doi:10.1371/journal.pone.0335412)
Supplement: S7 Table — (PDF) [file pone.0335412.s008.pdf]

S7 Table. Multivariable linear regression analysis between the natural logarithms of the urinary calcium-to-magnesium ratio and albumin-to-creatinine ratio

	Total	Men	Women
	β (95% CI)	β (95% CI)	β (95% CI)
Model 1	−0.041 (−0.084, 0.002)	−0.148 (−0.211, −0.085)	0.084 (0.026, 0.143)
Model 2	−0.075 (−0.119, −0.031)	−0.136 (−0.201, −0.071)	0.005 (−0.055, 0.065)

The urinary calcium-to-magnesium ratio and urinary albumin-to-creatinine ratio were transformed to the natural logarithm. The analytic population in this model comprised 6,282 individuals (3,078 men and 3,204 women). Model 1 was adjusted for age, survey area, current smoker, never or rarely drinking, regular exercise habit, fasting status, and energy intake (quartiles). Model 2 was further adjusted for body mass index, hypertension, diabetes, history of urinary tract stone, and estimated glomerular filtration rate.
