# Supplementary material for: Associations of calcium and magnesium intakes and their intake ratio with albuminuria in middle-aged and older adults
Source: PLoS One. 2025 Nov 26;20(11):e0335412. doi: 10.1371/journal.pone.0335412 (PMC12654892; doi:10.1371/journal.pone.0335412)
Supplement: S8 Table — (PDF) [file pone.0335412.s009.pdf]

S8 Table. Adjusted odds ratio (95% CIs) of microalbuminuria for the quartiles of the urinary calcium-to-magnesium ratio

Range	Case, n (%)	Model 1	*P* for trend	Model 2	*P* for trend
Total					
≥2.38	318 (20.3)	Reference	0.630	Reference	0.250
1.67–2.37	300 (19.2)	0.91 [0.76, 1.09]		0.94 [0.78, 1.13]	
1.06–1.66	303 (19.1)	0.87 [0.73, 1.05]		0.93 [0.77, 1.13]	
<1.06	369 (23.5)	1.06 [0.89, 1.26]		1.13 [0.93, 1.37]	
Men					
≥2.33	139 (18.0)	Reference	0.011	Reference	0.090
1.57–2.32	136 (17.8)	0.93 [0.71, 1.22]		0.94 [0.71, 1.23]	
0.96–1.56	156 (20.3)	1.05 [0.81, 1.37]		1.05 [0.80, 1.38]	
<0.96	201 (26.1)	1.34 [1.04, 1.73]		1.23 [0.94, 1.63]	
Women					
≥2.44	177 (22.1)	Reference	0.028	Reference	0.771
1.74–2.43	175 (21.8)	0.98 [0.77, 1.25]		1.01 [0.78, 1.30]	
1.16–1.73	143 (17.9)	0.75 [0.58, 0.96]		0.85 [0.65, 1.11]	
<1.16	163 (20.4)	0.82 [0.64, 1.05]		1.02 [0.78, 1.34]	

The urinary calcium-to-magnesium ratio was divided by quartile group. Microalbuminuria was defined as a urine albumin-to-creatinine ratio of 30 mg/g or more. The analytic population in this model comprised 6,282 individuals (3,078 men and 3,204 women). Model 1 was adjusted for age, sex, survey area, current smoker, never or rarely drinking, regular exercise habit, fasting status, and energy intake (quartile). Model 2 was further adjusted for body mass index, hypertension, diabetes, history of urinary tract stone, and estimated glomerular filtration rate.
